# Quantitative Proteomic Analysis of Cellular Responses to a Designed Amino Acid Feed in a Monoclonal Antibody Producing Chinese Hamster Ovary Cell Line

**DOI:** 10.29252/.22.6.385

**Published:** 2018-11

**Authors:** Fatemeh Torkashvand, Fereidoun Mahboudi, Manouchehr Vossoughi, Elnaz Fatemi, Seyed Masoud Moosavi Basri, Amir Heydari, Behrouz Vaziri

**Affiliations:** 1 Medical Biotechnology Department, Biotechnology Research Center, Pasteur Institute of Iran, Tehran, Iran; 2 Department of Chemical and Petroleum Engineering, Biochemical and Bioenvironmental Research Center Sharif University of Technology, Tehran, Iran; 3 Department of Chemical Engineering, University of Mohaghegh Ardabili, Ardabil, Iran

**Keywords:** CHO cells, Glutathione, Monoclonal antibodies, Proteomics

## Abstract

**Background:**

Chinese hamster ovary (CHO) cell line is considered as the most common cell line in the biopharmaceutical industry because of its capability in performing efficient post-translational modifications and producing the recombinant proteins, which are similar to natural human proteins. The optimization of the upstream process via different feed strategies has a great impact on the target molecule expression and yield.

**Methods:**

To determine and understand the molecular events beneath the feed effects on the CHO cell, a label-free quantitative proteomic analysis was applied. The proteome changes followed by the addition of a designed amino acid feed to the monoclonal antibody producing CHO cell line culture medium were investigated.

**Results:**

The glutathione synthesis, the negative regulation of the programmed cell death, proteasomal catabolic process, and the endosomal transport pathway were up-regulated in the group fed with a designed amino acid feed compared to the control group.

**Conclusion:**

Our findings could be helpful to identify new targets for metabolic engineering.

## INTRODUCTION

Chinese hamster ovary (CHO) cells are desired hosts for the production of biopharmaceuticals. Their ability to perform efficient post-translational modifications and to produce the recombinant proteins, which are similar to natural human proteins, making them the most common cell line in the biopharmaceutical industry. Moreover, these cells are simply adapted to grow in a serum-free medium[1,2]. The use of fully chemically defined serum-free media has simplified the downstream processing and minimized the batch-to-batch variations[3]. Over the past two decades, the development of CHO cells with a considerable production yield has mainly been related to the process engineering strategies so as to optimize the media and feeds formulation, new feeding strategies, and the culture conditions[4,5]. Other approaches to achieve a high titer of biopharmaceuticals in CHO cells are genetic engineering through different expression vector design[6], cell engineering via the manipulation of the genes associated with the cell cycle[7], cell metabolism[8], apoptosis[9,10], and the protein secretion pathway[11]. Until now, the achievement rate of the protein titer enhancement through the genetic and cell engineering has been little compared to the bioprocess optimization methods[5].

The full characterization of cellular machinery of the CHO cells will result in more knowledge regarding the improvement of the manufacturing processes[12]. Despite the advances in improving the bio-pharmaceuticals productivity of the CHO cells, the complete characterization of their cellular machinery has still to be achieved[13]. In order to reach the comprehensive understanding of the CHO cell physiology and subsequently, the profound knowledge of the biopharmaceuticals production, systems biology approach has been employed[14,15]. Furthermore, in order to understand the cellular mechanism of the high productivity in CHO cells, different systems biology tools like transcriptomics, proteomics, and metabolomics have been applied[16,17]. Previous investigations that used the systems biology tools have studied differences between the cell line productivity[18-22] and the changes in culture conditions, including the temperature shift[23], sodium butyrate treatment[24,25], and hyperosmolarity[26], which enhance the productivity. Previous proteomic studies have also provided rational strategies for the modifications needed to increase productivity, develop cell growth, improve media consumption, and provide constant glycosylation patterns[27-38].

It is important to comprehend the molecular rationale of the final production increase by the feed compounds. Such knowledge will help bring about the design of new generation feeds to engineer the new cellular processes[17,39]. In this study, by using the label-free quantitative proteomic analysis, protein expression profiles were investigated, followed by the addition of a designed amino acid feed. Through this comparative proteomic study, correlation of the key biological processes influenced by the designed feed, along with the enhancement of the target protein productivity in the CHO cell line were shown. To the best of our knowledge, this is the first proteomic report that considers the effect of feed formulation on the biological processes during the production of the target monoclonal antibody (mAb) in CHO cells.

## MATERIALS AND METHODS

### Cell line and culture media

Recombinant CHO cell producing Bevasizumab, a mAb against the vascular endothelial growth factor A, was kindly provided by Aryogen Pharmed (Alborz, Iran). The cell line original source was purchased from the Life Technologies (California, USA). The basal culture medium was CDM4CHO (Hyclone Laboratories, Utah, USA), which was supplemented with six mM L-glutamine (Lonza, Verviers, Belgium) at the moment of the preparation according to the manufacturer’s instruction.

### Culture conditions

The CHO cells were cultured in a 500-ml shaker flask with an effective volume of 100 ml, incubated at 37 °C with 5% CO_2_ and agitated at 80 rpm. In the middle of the logarithmic phase, a temperature shift to 32 °C was performed. Each shaker flask was inoculated with an approximate cell density of 5 × 10^5^ cells/ml.

### Feeds and their components

The feeds used in this study were a home-made designed amino acid feed (feed A) and a control feed (feed B). The home-made designed amino acid feed containing 75 mM aspartic acid, 15 mM glutamic acid, 2.5 mM glycine, and 12.5 mM arginine (HiMedia Laboratories, Mumbai, India) was prepared based on the design of experiment methods, reported in our previous study[40]. In summary, the critical amino acids for the enhancement of target mAb production were determined employing the Plackett-Burman design. After finding the critical amino acids, their concentration in the feed was optimized by response surface methodology using a Box-Behncken design. The amino acids were dissolved in the basal medium (CDM4CHO), as control feed, and all the feeds were added, as multiple, discrete additions to the culture on days 3, 5, and 7.

### Proteomic analysis

Three biological replicate cultures from each group cultured in shaker flasks were examined using the label-free quantitative proteomic analysis. The samples were harvested on day 10. A total of 30 million cells were collected from each replicate of the cell line. The cell suspensions were centrifuged at 180 ×g at 4 °C for 5 min. The cell pellets were washed three times with isotonic sucrose solution (250 mM). The cells were resuspended in lysis buffer (7 M of urea, 2 M of thiourea, 4% CHAPS 40 mM of Tris, 0.2% Biolyte 3/10, and 50 mM of dithiothreitol). The whole-cell extracts were kept at room temperature for 60 min, followed by centrifugation at 14,000 ×g at 4 °C for 15 min. The samples were digested by the filter-aided sample preparation method[41]. The digested peptides were suspended in 10 µl of 0.1% formic acid and subjected to nanoLC-MS/MS analysis.

Reverse-phase ultra-high pressure liquid chromate-graphy (UPLC) separation of the intracellular protein digests was performed on Easy-nLC1000 (Thermo Fisher Scientific, USA) equipped with an in-house made column (100 μm × 10 cm) packed with a reversed-phase ReproSil-Pur C18-AQ resin (3 μm, 120 Å, Dr. Maisch GmbH, Germany). A linear gradient from 0 to 50% mobile phase B (0.1% formic acid in 80% acetonitrile) was achieved against the mobile phase A (0.1% formic acid in water) over 50 min. LTQ-Orbitrap Elite mass spectrometry (Thermo Fisher Scientific) was used along with UPLC to acquire the mass and fragmentation information of the digested peptides.

RAW files were extracted using the Mascot version 2.3.02 (Matrix Science, London, UK) embedded into Proteome Discoverer 2.0 (Thermo Fisher Scientific). Mass spectrometry data were searched against UniProt, Chinese hamster sequence database (http://www.uniprot.org/). Parameters were set as follows: fully tryptic peptides with ≤2 missed cleavages were permitted; carbamidomethylation and oxidization were fixed and variable modifications, respectively: peptide mass tolerance was 10 ppm, and fragment mass tolerance was 20 mmu; the charge state of the peptides were set from +2 to +6. The cut-off of global false discovery rate for peptide and protein identification was set to 0.01.

Chromatographic peak intensity (peak area) was calculated. Protein species with at least two unique peptides were selected for protein species quantitation, and the relative quantitative protein ratios between the two samples were calculated by comparing the average of log2-transformed abundance values (three biological replicates). Student’s *t*-tests were performed to determine the significance of changes between samples. A fold-change ≥1.5 and *p* ≤ 0.05 were used as the thresholds to define differentially accumulated protein species.

### Bioinformatic analysis

Bioinformatic analysis of proteins was conducted according to Liu *et al*.[42]. Functional annotation and category analysis was carried out using an online software, Blast2GO (http://www.geneontology.org). A *p* ≤ 0.05 was used as the threshold to determine the significant enrichments of GO pathways.

### Western blot analysis

Western blot analysis was performed as described before in detail[43]. Aliquots of the protein samples (35 µg) were loaded on 12% SDS-PAGE. Subsequently, they were transferred to a nitrocellulose membrane using the Towbin buffer (25 mM of Tris, 192 mM of glycine, and 20% methanol) by a semi-dry Trans-Blot cell (Bio-Rad, USA), and transfer was verified by Ponceau S staining. The membrane was incubated in a blocking buffer (2.5% skim milk, 2.5% glycerol, and 0.05% Tween-20 in TBS) at 4 °C overnight. Furthermore, the membrane was rinsed in TTBS (100 mM of Tris–HCl, 0.9% NaCl, and 0.05% Tween-20, pH 7.5) for 10 min. It was then incubated for 2 h with a blocking solution containing primary antibodies: 1:10,000 rabbit monoclonal to glutathione synthetase (GSS), 1:1000 rabbit polyclonal to glucose-6-phosphate dehydrogenase (G6PDH), 1:1000 rabbit polyclonal to proteasome subunit beta (PSMB), and 1:1000 rabbit polyclonal to beta-actin (all from Abcam, USA). After washing three times for 5 min each with TTBS, the membrane was incubated again for 1 h in 1:500 horseradish peroxidase-conjugated anti-rabbit IgG secondary antibody (RayBiotech, Iran). The immunoreactive bands were then detected by ECL plus kit (GE healthcare, UK) using Kodak Image Station 4000MM Pro.

## RESULTS

**Fig. 1:**
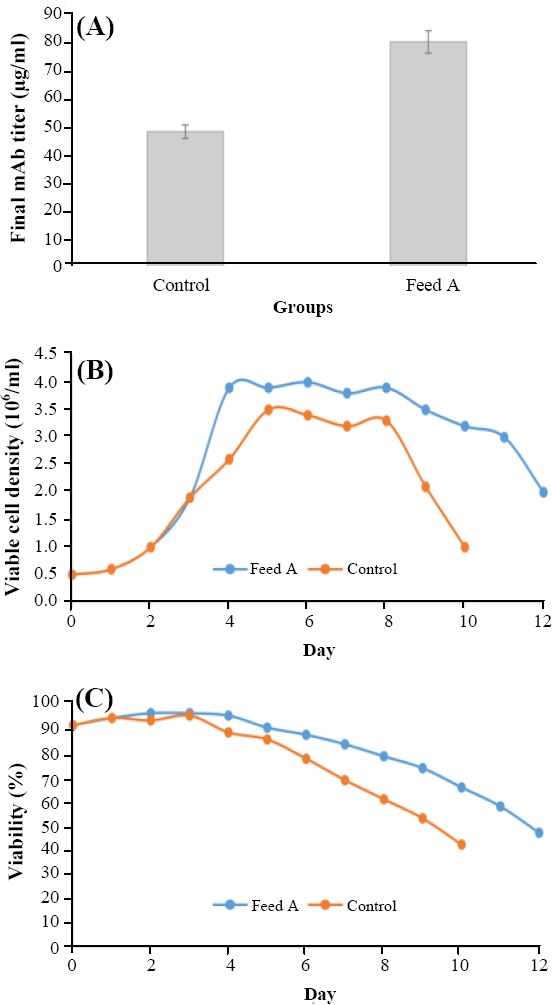
The final mAb titer (A), the viable cell density (B), and the viability percentage (C) in feed A group vs. control group.

The label-free quantitative proteomic analysis was incorporated to find the potential pathways and related gene targets to enhance the CHO cell productivity via the appropriate feeds. Comparative proteomics was performed on two groups: control and feed A. Three biological replicates were performed for each group, and the whole cell lysates from six shaker flasks were harvested on day 10 and further processed for the label-free analysis. The feeds were added as multiple discrete additions to the cultures on days 3, 5, and 7. In comparison to the control group, the final mAb titer increased by 70% in the group fed with feed A (Fig. 1A). Moreover, the viable cell density and viability percentage of the designed amino acid feed group improved (Fig. 1B and 1C).

### The label-free protein identification and the differential expression

The whole cell lysates from the biological replicates were harvested on day 10 and prepared for the label-free quantitative proteomic analysis. Label-free analysis results were provided as supplementary materials. On day 10, 41 proteins in the feed A group were differentially expressed in comparison with the control group. Among these proteins, 30 and 11 proteins were up-regulated and down-regulated, respectively in feed A group in comparison with the control feed group (Table 1).

**Table 1:** The list of differentially expressed proteins in feed A group

No.	Protein name	Protein ID	Fold change	Mus musculus homologous
1	Cystine/glutamate transporter	A0A098KXA0	3.2	Q9WTR6
2	Lactoylglutathionelyase	A0A061	2.4	Q9CPU0
3	Carboxylic ester hydrolase	A0A061IAA7	2.3	Q64176
4	Hydroxymethylglutaryl-CoA synthase, mitochondrial	G3HP76	2.3	P54869
5	Glutathione S-transferase	G3IFJ6	2.2	P30115
6	Bifunctionalmethylenetetrahydrofolate dehydrogenase/cyclohydrolase	A0A061HZ57	2.0	P18155
7	Plasminogen activator inhibitor 1	G3HA54	2.0	P22777
8	Transgelin	G3H7Z2	2.0	Q9WVA4
9	Extracellular matrix protein 1	G3H2W6	1.9	Q61508
10	Low-density lipoprotein receptor	P35950	1.9	P35951
11	Alpha-2-macroglobulin receptor-associated protein	G3GWB3	1.8	P55302
12	Hemeoxygenase 1	G3IAI6	1.8	P14901
13	4F2 cell-surface antigen heavy chain	G3IHN6	1.8	P108522
14	Macrophage migration inhibitory factor	G3HY08	1.8	P34884
15	Glutathione S-transferase	G3I5H1	1.8	P24472
16	Phosphoserine aminotransferase	G3IKH9	1.7	Q99K85
17	Macrophage metalloelastase	G3GUV3	1.7	Q63341
18	3 beta-hydroxysteroid dehydrogenase/Delta 5-->4-isomerase type 2	G3I6D1	1.7	P24815
19	Calponin	G3I3W0	1.7	Q9DAW9
20	von Willebrand factor A domain-containing protein 5A	G3IMX9	1.7	Q99KC8
21	Glucose-6-phosphate 1-dehydrogenase	O55044	1.6	Q00612
22	Guanine nucleotide-binding protein subunit gamma	G3H7D1	1.6	Q80SZ7
23	Prolyl 4-hydroxylase subunit alpha-1	G3HJ24	1.6	Q60715
24	CD44 antigen	P20944	1.5	P15379-2
25	Bone marrow stromal antigen 2	A0A061IGY1	1.5	Q8R2Q8
26	Galectin	G3H7B3	1.5	P16110
27	Glutathione peroxidase	G3HF60	1.5	O70325
28	EH domain-containing protein 1	G3I6F9	1.5	Q9WVK4
29	Glutathione synthetase	G3HAP7	1.5	P51855
30	Proteasome subunit beta type	G3H303	1.5	P99026
31	Lamin-B receptor	G3GRA0	-1.5	Q3U9G9
32	UDP-N-acetylhexosaminepyrophosphorylase-like protein 1	G3IJB9	-1.5	Q3TW96
33	Lon proteasehomolog, mitochondrial	G3HCJ1	-1.6	Q8CGK3
34	Translocon-associated protein subunit gamma	G3H223	-1.6	Q9DCF9
35	Mitochondrial-processing peptidase subunit alpha	G3I6Z3	-1.6	Q9DC61
36	NAD(P) transhydrogenase, mitochondrial	G3HMX6	-1.7	Q61941
37	Periaxin	A0A061I2N6	-1.7	unreviewed
38	Protein unc-84-like B	G3I2J5	-1.7	Q8BJS4
39	Protein AHNAK2-like protein	A0A061I4K9	-1.9	O55103-2
40	NADH-cytochrome b5 reductase	G3H3L4	-2.1	Q9DB73
41	CDGSH iron sulfur domain-containing protein 1	G3H4L8	-2.8	Q91WS0

### The up-regulation of important biological processes

The differentially expressed proteins were identified and clustered by the biological process using *Mus musculus* homologues and subjected to gene enrichment analysis by the gene ontology consortium. The significant clusters that were up-regulated in feed A group in comparison with the control feed group are presented in Table 2. The pentose-phosphate shunt, the glutathione (GSH) metabolic process, the negative regulation of the programmed cell death, the cellular response to the oxidative stress, the regulation of intracellular transport, and the proteasomal protein catabolic process were up-regulated in the group fed with feed A, in comparison with the control group. There was no significant biological process for the down-regulated proteins in feed A.

**Table 2:** Up-regulated significant biological processes in feed A

No.	Biological process	*p* value	GO ID
1	Pentose-phosphate shunt	0.01447	0006098
2	NADPH regeneration	0.01558	0006740
3	Glutathione metabolic process,	0.000001697	0006749
4	Negative regulation of programmed cell death (apoptotic signaling pathway)	0.00001436	2001234
5	Carboxylic acid biosynthetic process	0.01058	0046394
6	Cellular response to oxidative stress	0.02598	0034599
7	Regulation of vesicle-mediated transport	0.001639	0060627
	Regulation of intracellular transport	0.02796	0032386
8	Proteasomal protein catabolic process	0.02336	0010498

GO, gene ontology

### Western blot analysis result

**Fig. 2:**
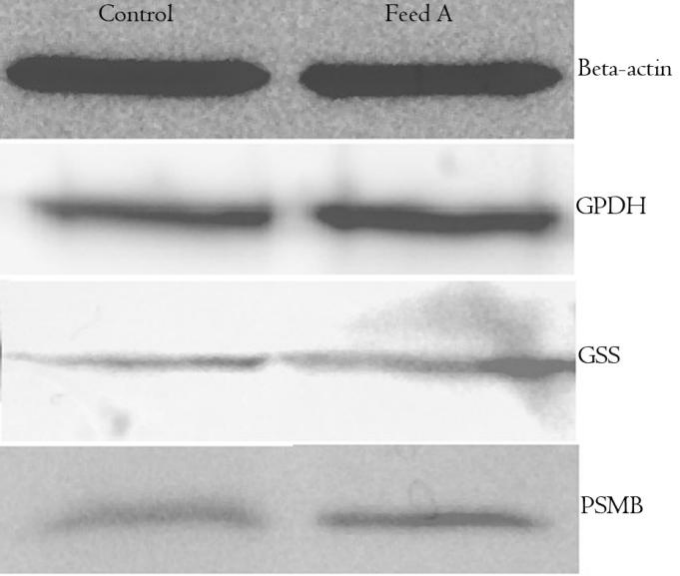
Western blot images of beta-actin, G6PDH, GSS, and PSMB of control group and designed amino acid feed group (feed A). Beta-actin was used as a control protein.

Western blot analysis of some of the differentially expressed proteins confirmed all the alterations identified by LC-MS/MS (Fig. 2). The selected proteins for Western blot analysis belonged to different biological processes. G6PDH, GSS, and PSMB were from pentose phosphate shunt, GSH metabolic process, and proteasomal protein catabolic process, respectively.

## DISCUSSION

The up-regulation of GSH pathway was identified in feed A group as compared to the control feed group. GSH is a significant antioxidant and plays several important roles in cells. GSH synthesis occurs in two steps: (1) glutamatecysteine ligase condensates cysteine and glutamate, and (2) a glycine is added by GSS to form GSH[44]. It was found that GSS was up-regulated in feed A on day 10 and was confirmed by Western blot analysis (Fig. 2). The up-regulation of this protein in the high-producing cell line has also been reported by Orellana *et al*.[19].

**Fig. 3:**
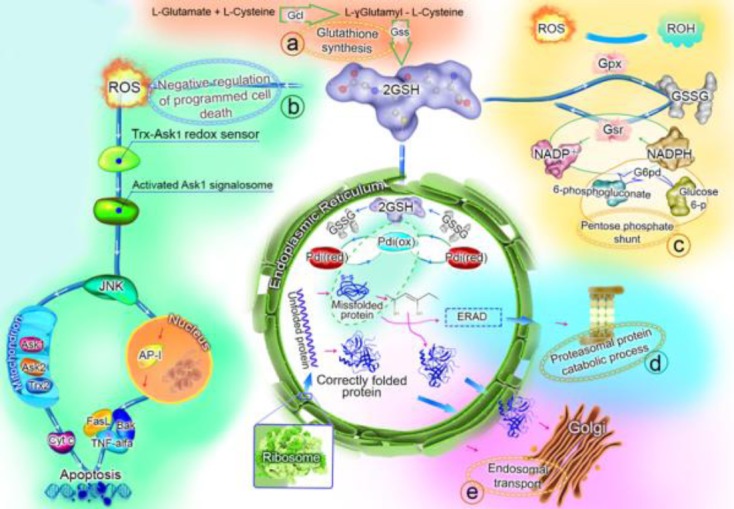
The suggested functions for the up-regulated biological processes in feed A group on day 10. The GSH synthesis (a), the negative regulation of programmed cell death (b), the pentose phosphate shunt (c), the proteasomal protein catabolic process (d), and the endosomal transport (e) were up-regulated processes in feed A group compared with the control feed group. The details and the roles of these processes in the final mAb titer enhancement have been explained in the text. GSH, glutathione (oxidized form); GSSG, glutathione (reduced form); Gcl, glutamatecysteine ligase; GSS, glutathione synthetase; Gpx, glutathione peroxidase; Gsr, glutathione reductase; G6pd, glucose-6-phosphate 1-dehydrogenase; Pdi, protein disulfide-isomerase; ERAD, endoplasmic reticulum-associated protein degradation; ROS, reactive oxygen species.

GSH mediates the correct protein disulfide bond formation by reducing the incorrect protein disulfide bonds via the protein disulfide-isomerase in the endoplasmic reticulum (ER)[45,46]. Furthermore, GSH can be oxidized to GSSG in order to detoxify the reactive oxygen species (ROS), which may result in the down-regulation of apoptosis (Fig. 3), and then GSSG can be reduced once more using nicotinamide adenine dinucleotide phosphate (NADPH) to complete the GSH redox cycle[47,48]. NADPH can be provided by the pentose phosphate shunt, which was also up-regulated in feed A group in comparison with the control group (Fig. 3). Moreover, G6PD catalyzes the rate limiting step of the pentose phosphate shunt, and the main function of this protein is to provide NADPH[49]. G6PD was also up-regulated in feed A on day 10 and was confirmed by Western blot analysis (Fig. 2). The up-regulation of GSH in the high mAb producing CHO cells has also been reported in previous studies[19,20]. Two main causes of high oxidative stress in the high-producing CHO cells are: the stress from the protein folding in ER and the stress from the mitochondrial respiration[20]. Recent studies have proposed some strategies to mAb-producing CHO cells for the metabolic engineering of the GSH preservation[50,51].

In the present proteomic analysis, the overexpression of proteins, which were involved in the negative regulation of apoptosis, was found. Furthermore, the batch duration and viability percentage were in line with this event, and the batch duration of feed A group was more than that of the control feed group (Fig. 1B and 1C). The cell apoptosis may occur in a fed-batch culture because of the hyperosmolarity, the by-product accumulation, and the production of ROS[52,53]. The use of a suitably designed feed can delay the apoptosis and extend the culture duration, which leads to an increase in the mAb productivity[54]. The negative regulation of the programmed cell death has been attributed to the role of GSH in the reduction of ROS[47], which also seems to be true in this study (Fig. 3). Based on the results obtained from this study, it seems that increase in productivity is due to increased integral viable cell density and somewhat to increased specific productivity.

In the recombinant protein production process, the protein translation and secretion are considered as bottlenecks[55,56]. There are different transport vesicles in cells, and the up-regulation of the proteins that constitute these vesicles has already been reported, as a result of recombinant protein overexpression[57]. The up-regulation of intracellular protein transport in the high mAb-producing CHO cells has also been reported by Orellana *et al*.[19]. In line with these reports, the present research demonstrates that the proteins involved in the intracellular transport and the vesicle-mediated transport such as the transitional ER ATPase, filamin A, and AP-2 complex subunit mu are up-regulated after the addition of appropriate feed. AP-2 is involved in the endocytosis and the transport from the trans-Golgi network to the lysosomes[58]. Another report has also showed the up-regulation of AP-2 in the high mAb producing cells[19].

If the protein production in cells increases, the misfolded proteins rate will also increase[59]. In order to prevent the accumulation of misfolded proteins, their retrotranslocation from ER to cytosol, when they undergo the ubiquitination, will occur, and these misfolded proteins will be the substrates for proteasome[60]. In addition, the up-regulation of the proteasomal catabolic process was seen in feed A, and the up-regulation of proteasome subunit beta was confirmed by Western blot analysis. This study shows that the feed composition will affect mAb production by changing the different pathways regulation, which are likely correlated to culture productivity.
